# Primary Psoas Muscle Hydatid Cyst: A Case Report of a Rare Pathology

**DOI:** 10.7759/cureus.76826

**Published:** 2025-01-02

**Authors:** Rahul K Ramtohul, Hosam Alazazzi, Muneeb Ullah, Amal N Qureshi, Colin T O'Connor, Ahmed Algahiny, Parisa Aquilini, Hussain Sajwani, Akashnath K Ganeshanath

**Affiliations:** 1 General Surgery, Beaumont Hospital, Dublin, IRL; 2 General surgery, Wrightington, Wigan and Leigh NHS Foundation Trust, Wigan, GBR; 3 General Surgery, Maroof International Hospital, Islamabad , PAK; 4 General Surgery, Rashid Latif Medical College, Lahore, PAK; 5 Medicine, University of Minnesota, Minnesota, USA; 6 Internal Medicine, University Hospitals of Leicester NHS Trust, Leicester, GBR; 7 Internal Medicine, Royal College of Surgeons in Ireland, Dublin, IRL; 8 General Surgery, Royal College of Surgeons in Ireland, Dublin, IRL; 9 General and Colorectal Surgery, NHS Wales, Wales, GBR

**Keywords:** echinococcus granulosus, hydatid cyst, lower back, psoas muscle, rare location

## Abstract

While hydatid cysts typically involve the hepatic system, extra-hepatic manifestations have rarely been reported. Of these cases, psoas muscle involvement remains exceedingly rare. Hydatid cyst in unusual locations is often found incidentally. In addition to surgery, which is the primary treatment for hydatid illness, anti-parasitic drugs known as benzimidazoles should also be used to treat hydatid cysts. The numerous sites and possible problems must be known to the treating surgeons. Pre-operative radiography is essential because it aids in the detection of the cyst, allows for its assessment as a differential diagnosis, and defines the treatment plan to prevent fatal outcomes. We describe an interesting case of a psoas hydatid cyst with an esoteric surgical approach.

## Introduction

The larvae of the parasite *Taenia Echinococcus granulosus* affect humans by causing hydatid disease [1]. The definitive host for this parasite is the dog, as well as other predatory animals like wild canines. By consuming Taenia eggs in contaminated food and water, humans unintentionally become the intermediary hosts [2,3]. The liver (50-70%) and lungs (20-30%) are the most commonly affected organs, whereas other organ involvement is uncommon (10%) [4]. Epigastric or right hypochondriac pain, nausea, and vomiting are common symptoms that appear gradually. More than 70% of patients have a single cyst, and 85-90% of cases only affect one organ. Cysts may eventually put pressure on adjacent tissues depending on their size and location, causing discomfort and agony in the abdomen [4,5]. Although this condition is preventable and treatable, it continues to be a major public health concern, especially in hydatid cyst-endemic countries [6]. Cystic echinococcosis disease can be eradicated in fewer than 10 years by taking preventive measures, which include vaccination of lambs, deworming of dogs with Praziquantel, increased sanitation during the slaughter of livestock, and public education campaigns. Hydatid cyst is distributed globally, with an annual incidence rate of 1-200 per 100,000 people. For surgical patients with cystic echinococcosis, the average postoperative mortality rate is 2.2%, and roughly 6.5% of cases relapse after an intervention, necessitating a lengthy recovery period [3]. In this case report, a patient from a region where echinococcus is endemic presents with an unexpected swelling in the left lower back, making the differential diagnosis of hydatid cyst challenging. Just 2% to 3% of hydatid cyst instances are found in the psoas muscle, making it an extremely uncommon site [1]. Thus, the case presented here is unique due to its peculiar location in the left psoas muscle.

## Case presentation

A 50-year-old male presented to the outpatient department with complaints of swelling and pain in his left lower back that had persisted for the past five years. The onset was gradual, with symptoms progressively worsening over time. The patient described the pain as persistent and intensifying both over time and with movement. On admission, he was hemodynamically stable. Routine investigations revealed leucocytosis, a hemoglobin level of 10.5 mg/ml, and a CRP level of 13.5 mg/dl. Additional diagnostic tests, such as echinococcus IgG Enzyme-Linked Immunosorbent Assay (ELISA), countercurrent immunoelectrophoresis (CIEP), coagglutination, and latex agglutination, were not performed.

The patient, originating from an echinococcus-endemic region, had previously been diagnosed with a tuberculous cold abscess in the psoas muscle. After initiating an eight-week course of anti-tuberculosis therapy consisting of isoniazid, rifampin, pyrazinamide, and ethambutol, his fever resolved. However, subsequent CT/MRI imaging demonstrated a progressive increase in the size of the lesion, with dimensions expanding from 3x1x2.5 cm to 4x3x2.8 cm (approximately 1x2x0.3 cm increase) compared to CT images eight months prior. Despite the resolution of the fever, his abdominal pain and swelling persisted and gradually worsened, correlating with the progression observed in imaging. As a result of the worsening symptoms, adjustments in treatment were considered, but no new interventions were initiated at this stage.

Investigations

**Figure 1:**
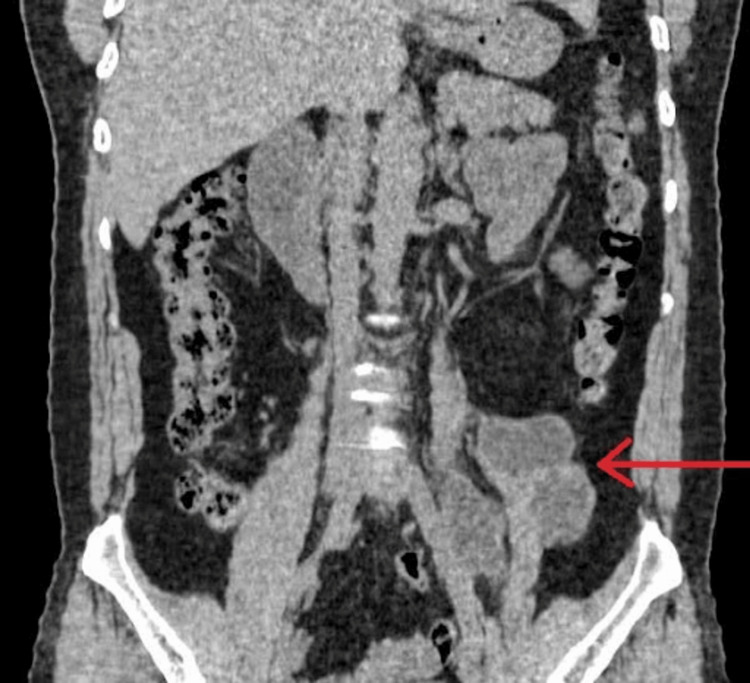
CT scan without contrast. Coronal view (red arrow) showing multiseptated left psoas cyst.

**Figure 2:**
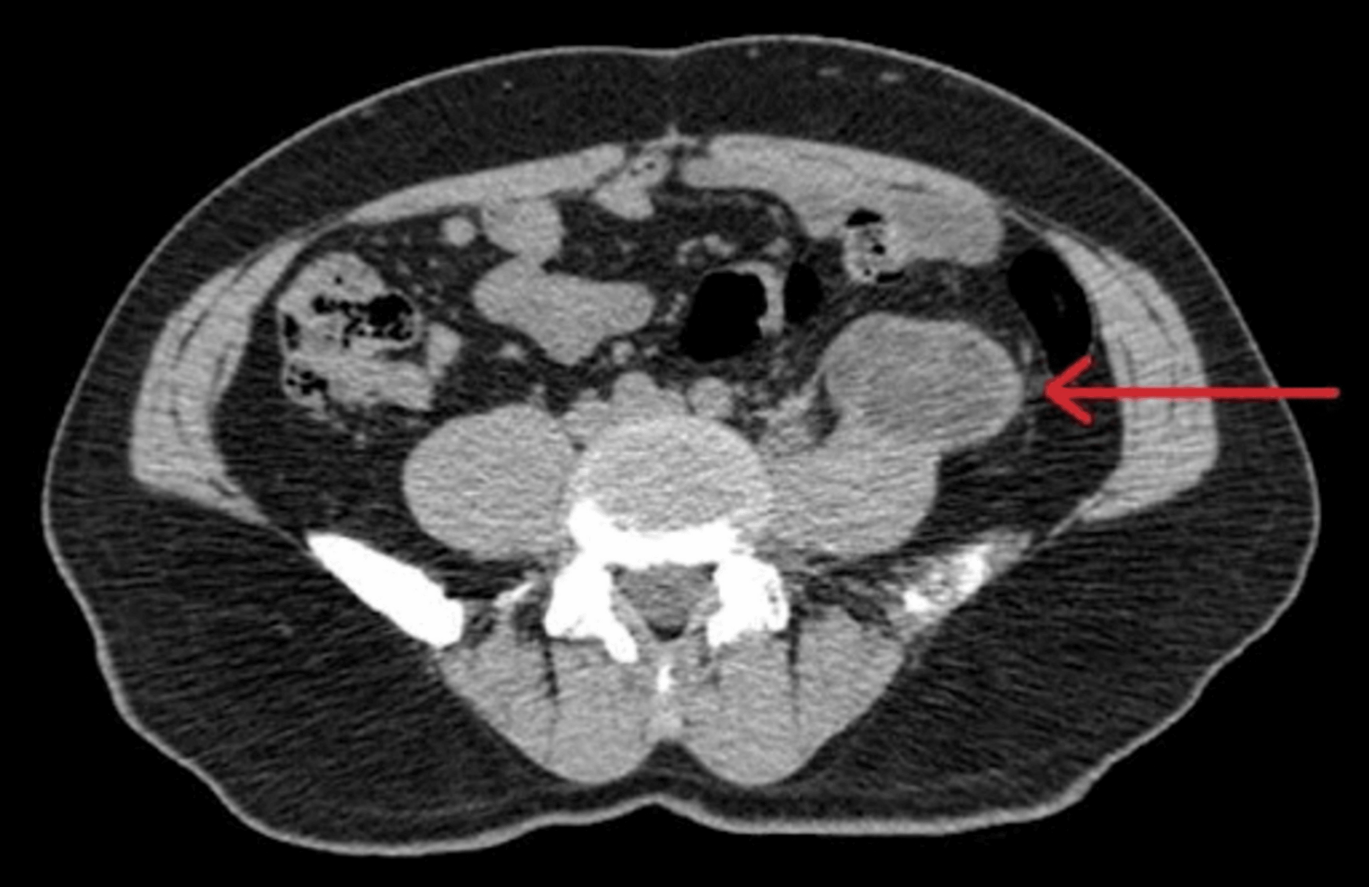
CT scan without contrast. Axial view (red arrow) showing multiseptated left psoas cyst.

CT (Figures 1-2) revealed multiloculated collection along the anterior, medial & lateral aspects of the middle and distal one-third of the left psoas muscle. It measured 4 cm in craniocaudal (CC), 3 cm in anterior-posterior (AP), and 3 cm in transverse (TR) dimensions. Medially, it extends deep to the medial margin of the psoas muscle and causes anteromedial displacement of the left ureter, and laterally, it causes left lateral displacement of the left psoas muscle. Posteriorly, it is inseparable from the anterior margin of the psoas muscle.

MRI revealed a multiloculated septate peripherally enhancing collection/abscess along the anterior aspect of the left psoas muscle deep to its fascia, which has a bulging exophytic component laterally in the left iliac fossa measuring approximately 30×70 mm in TR and AP dimensions.

Preoperative

The lesion was initially managed as a multiloculated tuberculous cold abscess with an eight-week course of anti-tuberculosis therapy consisting of Isoniazid, Rifampin, Pyrazinamide, and Ethambutol. Open drainage was planned; however, perioperative aspiration revealed clear fluid, leading to the diagnosis being revised to a hydatid cyst. Consequently, antimicrobial therapy with Ceftriaxone was initiated.

Intraoperative

A total cystectomy with deroofing was performed using an open surgical approach, considering the complexity of the anatomical location and the size of the cyst. This was complemented with albendazole therapy. Repetitive cycles of irrigation and drainage were done, followed by the placement of a Jackson-Pratt drain. Specimens were collected in an endo bag and sent for histopathology examination. The procedure was completed successfully without any complications.

Histopathology

Gross

The specimen is received in formalin fixative and comprises multiple already cut-opened-cysts, the largest measuring 5x3x0.6 cms and the smallest measuring 1.5x1.0x0.6 cms. The cyst wall is comprised of soft gelatinous greyish-white tissue, and its thickness is 0.1 cm. Representative sections are submitted in two cassettes.

Histology

Excision biopsy of the cyst (left psoas) revealed sections of the cyst wall with a lining composed of an inner germinal layer and an outer chitinous, acellular, lamellate layer, consistent with an echinococcus cyst (hydatid cyst). Unfortunately, we do not have histological images to include here at this time.

Postoperative

At discharge, the patient was given albendazole 200 mg x 2 tablets x PO x BD for 30 days. This 30-day regime was repeated after a 14-day treatment-free period for a total of three consecutive cycles. The patient was also prescribed flagyl 800 mg TDS for seven days, along with ceftriaxone 1 gm BD for three days. 

The first follow-up was after three days, and the second was after two weeks. Due to the usage of albendazole, liver function tests were monitored at each follow-up and were found to be normal. The patient was responding satisfactorily. No recurrences were found on follow-up.

## Discussion

Echinococcosis, commonly known as hydatidosis, is a potentially lethal disease caused by cysts harboring the larval stages of the *Echinococcus granulosus* tapeworm (Dog Tapeworm) [4]. Hydatid disease affects pulmonary and hepatic systems in 80-90% of the cases [7] and rarely affects the psoas muscle, accounting for 1-3% involvement. Hydatid cyst in unusual locations is often found incidentally. Occasionally (2-3% each), symptomatic cysts in the kidney, spleen, peritoneal cavity, skin, and psoas muscles have been recorded [8-10]. The psoas muscle serves as an unfavorable environment for embryophore development due to muscle contraction leading to subsequent lactic acid generation, which explains the rarity of this localization [9,11]. 

The exact pathophysiology explaining the rarity of psoas muscle involvement still remains unknown. However, few authors assert that direct inoculation of the embryo can be either through a dog bite or through direct systemic circulation after leaving the gut and passing through hepatic and pulmonary systems [9,11]. According to Ergul et al., the bulk of the muscle mass and its abundant blood supply may be responsible for the predominance of the concentration of hydatid larvae in the proximal muscles of the lower limbs [12]. This exceptional nature of involvement of the psoas muscle typically leaves the patient asymptomatic until it compresses the surrounding structures, including kidneys, ureters, and the vertebral body [13]. Around thirty cases have been reported, which highlights the rarity of the psoas muscle involvement in hydatid disease [12,14]. 

The yearly growth rate of the cyst ranges from 1 to 3 cm. The cysts might have thin or thick walls with one or several loci or single or multiple in number. Upon evaluation of the involved soft tissues, we found that these cysts frequently show clinically palpable masses. The hydatid disease's clinical course is not known with certainty. It is dependent upon a number of factors, including quantity, size, and location of the cysts. A wide range of differential diagnoses should be taken into account when suspecting hydatid disease due to its rare clinical manifestation. These differentials include an abscess, synovial cyst, necrotic malignant soft tissue tumor, tuberculosis and persistent hematoma, hydronephrosis, and pancreatic cyst [12,13,15]. The combination of a number of factors, such as patient origin, clinical symptoms, imaging, and serology, is used to confirm the diagnosis [15]. 

Ultrasound is a helpful imaging technique that may be utilized for the diagnosis, classification, and follow-up of hydatid cysts even if it lacks the sensitivity to detect tiny cysts. Moreover, it offers crucial details on cyst size and location, adjacency of cysts to anatomical structures, cyst count, and cyst component characteristics [16]. In addition to the US, magnetic resonance imaging (MRI) and computed tomography (CT) can be utilized to look for additional organ involvement. A highly helpful imaging method for making a conclusive diagnosis, CT catches cysts under 1 cm in size and may be utilized to assess every organ. The accuracy rate of CT diagnosis has been listed as 61-96% in the literature [17,18]. When there is no obvious clinical picture and imaging evaluation is insufficient for a conclusive diagnosis in the presence of a sterile cyst, serological testing may be utilized [19]. In our instance, serological testing wasn’t necessary. Ultrasound abdomen typically reveals a cystic lesion which is occasionally calcified with daughter cysts, with or without membrane separation or a multi-layered wall of the cyst, calcified or thick wall of the cyst or pseudo-tumoral according to the emerging stage of the hydatid illness [11]. 

Despite the use of anti-parasitic medications known as benzimidazoles for the eradication of hydatid cysts [8], this treatment should be adjunct to surgery which is the mainstay treatment of hydatid disease. The preferred method is a total cystectomy without contamination of the area [15]. The type of surgery required is often dependent on the cyst's size and location. Although it can injure the psoas muscle, drastic surgery such as complete excision is undertaken on cysts smaller than 5 cm in size. In contrast, bigger cysts are treated with a more cautious approach by sectioning the projecting dome due to a higher risk of complications. Though surgical intervention carries a risk of complications including ruptured cysts that may result in residual hematic/ purulent effusions and carry the risk of recurrence, the overall prognosis remains generally favorable [11]. Which is what we opted for due to anatomical complexity and risk of recurrence. Given the anatomical complexity and risk of recurrence in this case, we opted for this approach.

Alternatively, percutaneous drainage using the PAIR (Puncture, Aspiration, Injection, and Re-aspiration) technique is a favorable option for anatomically suitable lesions, such as those in the liver. PAIR has become the primary treatment method for lesions that are both anatomically and surgically accessible, boasting a success rate of over 75% in treating hydatid cysts. During the procedure, patients receive prophylactic albendazole to enhance efficacy and reduce the risk of recurrence. Scolicidal agents such as hypertonic (20%) saline, 0.5% cetrimide with 0.05% chlorhexidine, absolute alcohol, and others are utilized. Among these, 20% alcohol has demonstrated 100% scolicidal action after just six minutes of contact.

For instance, Bedioui et al. documented nine cases with a 2:1 male-to-female ratio [10]. Seven patients underwent the extraperitoneal Leriche approach, while two had transperitoneal median laparotomy. In all cases, partial cystectomy was performed, preserving pericystic tissue near neurovascular structures. No operative mortality was reported, and only one patient developed a postoperative urinary infection. During 2.5 years of follow-up, there was a single case of recurrence four years after surgery, requiring reoperation [10].

The unusual positioning of hydatid cysts often gives rise to complications linked to their anatomical location. Reported complications include femoral nerve damage, ureteral compression, and kidney dysfunction caused by compression. In more severe cases, patients may develop sepsis or face an increased risk of shock [20].

## Conclusions

Due to its rare location, this case is quite remarkable. To make an accurate differential diagnosis of a cystic painless mass in every anatomical region and to better manage their patients, treating surgeons must be informed of the many locations and potential problems that are concomitant with hydatid cysts. Failure to recognize hydatid cysts in unusual locations, such as the psoas muscle, can result in suboptimal management, seeding infectious, and poor operative outcomes. Preoperative radiography is imperative since it assists in locating the cyst, allowing for its consideration as a differential diagnosis and establishing the management strategy in order to prevent fatal complications.
